# Hydrotherapy

**Published:** 1910-11

**Authors:** 


**Hydrotherapy.** A treatise on hydrotherapy in general; its application to special affections; the Technic or Process Employed; and use of Waters Internally. By **Guy Hinsdale**, A.M., M.D., Lecturer on Climatology, Medico-Chirurgical College of Philadelphia. Octavo of 466 pages. Illustrated. Philadelphia and London: W. B. Saunders Company, 1910. (Cloth, $3.50 net.)

To this author and to Dr. Simon Brauch the medical profession of the United States owe a debt of gratitude for so greatly advancing the technic and practice of hydrotherapy. It is only within the last fifty years that a scientific system and scientific application has been evolved.

Many diseases are amenable to hydrotherapeutic treatment if guided along rational lines. In order that this may be done the physical and chemical qualities of water should be clearly understood. Dr. Hinsdale has issued a good work on this subject. J. A. R.

